# Altered functional connectivity in blepharospasm/orofacial dystonia

**DOI:** 10.1002/brb3.894

**Published:** 2017-12-18

**Authors:** Angela Jochim, Yong Li, Gina Gora‐Stahlberg, Tobias Mantel, Maria Berndt, Florian Castrop, Christian Dresel, Bernhard Haslinger

**Affiliations:** ^1^ Department of Neurology Klinikum rechts der Isar Technische Universität München Munich Germany; ^2^ Department of Neuroradiology Klinikum rechts der Isar Technische Universität München Munich Germany; ^3^ Department of Neurology and Neurologic Rehabilitation Asklepios Stadtklinik Bad Tölz Bad Tölz Germany; ^4^ Department of Neurology School of Medicine Johannes Gutenberg University Mainz Germany

**Keywords:** blepharospasm, functional connectivity, Meige's syndrome, orofacial dystonia, resting‐state functional MRI

## Abstract

**Introduction:**

Blepharospasm is characterized by involuntary eyelid spasms. It can be associated with perioral dystonia (Meige's syndrome or orofacial dystonia). We aimed at studying resting‐state functional brain connectivity in these patients and its potential modulation by therapeutic botulinum toxin injections.

**Methods:**

We performed resting‐state functional MRI and a region of interest‐based analysis of functional connectivity in 13 patients with blepharospasm/Meige's syndrome in comparison to 13 healthy controls. Patients were studied before and 4 weeks after botulinum toxin treatment. Simultaneous facial electromyography was applied to control for involuntary facial movements.

**Results:**

Before botulinum toxin treatment, patients showed reduced functional connectivity between caudate and primary sensorimotor, somatosensory association and visual cortices as well as between putamen and parietal association cortex. Cerebellar areas displayed decreased functional connectivity to somatosensory and visual association cortices. On the cortical level, connectivity was reduced between the cingulate cortex and the primary sensorimotor/premotor and parietal association cortex, between premotor areas and the primary somatosensory cortices, and between the postcentral gyrus and temporoparietal, secondary somatosensory, cingular, and cerebellar regions. Botulinum toxin treatment modulated functional connectivity, especially between cerebellum and visual cortices.

**Conclusions:**

Patients with blepharospasm/Meige's syndrome show altered functional connectivity at rest in widespread brain regions including basal ganglia, cerebellar, primary/secondary sensorimotor, and visual areas. Functionally, this may reflect a predisposition for defective movement inhibition and sensorimotor integration. Botulinum toxin treatment could modulate brain connectivity in blepharospasm by altering visual and sensory input.

## 1 INTRODUCTION

Blepharospasm is a focal dystonia with involuntary bilateral eyelid spasms and increased blinking rate. When accompanied by perioral dyskinesia, it is called Meige's syndrome or orofacial dystonia (OFD) (Colosimo, Suppa, Fabbrini, Bologna, & Berardelli, 2010). Local injection of botulinum toxin (BTX) represents the most effective therapy for this condition.

The pathophysiology of OFD is not well understood. Functional magnetic resonance imaging (fMRI) showed a reduced activation of the primary motor and ventral premotor cortex during an oral motor task in OFD patients compared to healthy controls (HCs) and to patients with isolated blepharospasm. Activation of bilateral somatosensory and supplementary motor areas was increased in both patient groups (Dresel, Haslinger, Castrop, Wohlschlaeger, & Ceballos‐Baumann, 2006) and deficient activation in primary and secondary somatosensory representations of affected and unaffected body regions was demonstrated during somatosensory stimulation in OFD patients (Dresel et al., 2011). Others found enhanced activation of anterior cingulate cortex, primary motor cortex, thalamus, and cerebellum during voluntary blinking in blepharospasm patients (Baker, Andersen, Morecraft, & Smith, 2003). Together, these findings were interpreted in the context of impaired (cortical) inhibition and changes in somatosensory representations in analogy to previous findings in other forms of focal dystonia (Neychev, Gross, Lehericy, Hess, & Jinnah, 2011).

FMRI at rest (rsfMRI) allows studying changed interaction between brain areas (functional connectivity [FC]; Fox & Raichle, 2007), possibly underlying the emergence of clinical symptoms independent from any task. Some previous resting‐state (rsfMRI) studies in focal hand dystonia showed altered FC within the sensorimotor network (Delnooz, Helmich, Toni, & van de Warrenburg, 2012; Dresel et al., 2014; Mohammadi et al., 2012). Two rsfMRI trials on blepharospasm analyzed the amplitude of low‐frequency fluctuations (ALFF) and showed changes in the striatum, thalamus, cerebellum, somatosensory regions, (orbito)frontal/prefrontal cortex as well as cingulate and insular cortex (Yang et al., 2013; Zhou et al., 2013). While ALFF analyzes resting‐state brain activity on the voxel level, the analysis of FC between various brain regions seems to be of special interest considering the emerging concept of dystonia as a network disease (Neychev et al., 2011). We therefore examined patients with isolated and idiopathic dystonia, namely blepharospasm with or without mild OFD using rsfMRI before and after BTX treatment in comparison with HCs. Simultaneous electromyographic (EMG) recording was applied to control for involuntary facial movements during MRI.

## 2 MATERIALS AND METHODS

### 2.1 Subjects

Thirteen patients (five women) and thirteen HCs, matched for gender and age, were studied (Table 1). All subjects were screened for neurologic and psychiatric diseases as well as previous neuroleptic medication, which were negative with the exception of one patient with poliomyelitis in childhood with residual mild paresis of the right foot and one HC with polyneuropathy.

**Table 1:** Subjects’ characteristics

	Patients		Healthy controls	*p*
Age (years; mean ± *SD*)	62.3 ± 11.2		63.5 ± 11.7	.85^a^
Gender (male/female)	9/4		9/4	1
Diagnosis (blepharospasm/Meige′s)	9/4		—	—
Disease duration (years; mean ± *SD*)	9.3 ± 7.0		—	—
Interval BTX—post‐BTX MRI (days; mean ± *SD*)	30.1 ± 3.8		—	—
Interval BTX—pre‐BTX MRI (days; mean ± *SD*)	87.8 ± 12.5		—	—
BTX treatment				
Abobotulinum toxin (*n*; mean of dose ± *SD*)	6; 165 ± 34.3		—	—
Onabotulinum toxin (*n*; mean of dose ± *SD*)	5; 33.75 ± 8.51		—	—
Incobotulinum toxin (*n*; mean of dose ± *SD*)	2; 63.75 ± 11.25		—	—
	**Pre‐BTX**	**Post‐BTX**		
Blepharospasm disability scale (mean ± *SD*)	0.54 ± 0.24	0.63 ± 0.28	—	.109^b^
Jankovic rating scale (mean ± *SD*)	4.1 ± 2.6	3.4 ± 2.2	—	.413^b^
Number of blinks during rsfMRI (642.6 s) detected by electromyography (mean ± *SD*)	277.4 ± 292.7	276.0 ± 346.3	233.3 ± 181.4	.66^c^.70^d^.97^e^

Blepharospasm/OFD patients and healthy controls were matched in respect of gender and age (±5 years).

BTX, botulinum toxin; pre‐/post‐BTX, before/after BTX treatment; rsfMRI, resting‐state functional magnetic resonance imaging; *SD*, standard deviation.

a Independent‐sample *t* test.

b Wilcoxon signed‐rank test.

c pre‐BTX versus HC; independent‐sample *t* test.

d post‐BTX versus HC; independent‐sample *t* test.

e pre‐BTX versus post‐BTX; paired *t* test.

Nine patients suffered from isolated blepharospasm, while four had additional mild orofacial symptoms without relevant dyskinesia at rest (mean disease duration 9 ± 7 years, Table 1). All patients were treated with BTX type A injections in the periorbital region (mean duration 6 ± 7 years, intertreatment interval ≥12 weeks). As the perioral symptoms of OFD patients were only mild, BTX was only injected in the periorbital region. The patients were studied twice, at the expected peak of the BTX effect, 4 weeks after treatment (post‐BTX, on average 30.1 ± 3.8 days after treatment) and about twelve weeks after treatment, when the main BTX effect had waned (pre‐BTX, on average 87.8 ± 12.6 days after treatment). The order of scanning sessions (pre‐/post‐BTX) was pseudorandomized.

All subjects were right handed as tested by the Edinburgh Handedness Inventory (Oldfield, 1971). The patients were recruited from the outpatient clinic for movement disorders of the Department of Neurology, Klinikum rechts der Isar, Technische Universität München. Written informed consent according to the Declaration of Helsinki was obtained by all subjects, and the study protocol was approved by the local ethics committee.

### 2.2 Clinical assessments

Before scanning, all patients were videotaped according to the Dystonia Study Group Videotape examination protocol (Burke et al., 1985). The videotapes were used for the exclusion of dystonic symptoms exceeding orofacial dystonia and for a blinded rating by an experienced movement disorders specialist (B.H.) using the Jankovic Rating Scale (JRS) (Jankovic, Kenney, Grafe, Goertelmeyer, & Comes, 2009). Additionally, the patients were asked to rate their subjective blepharospasm‐related disability using the Blepharospasm Disability Scale (BDS; Lindeboom, De Haan, Aramideh, & Speelman, 1995). To screen for gross cognitive deficits, we applied the multiple‐choice word test (MWTB) (Table 1; Lehrl, 2005).

### 2.3 Facial electromyography

**Figure 1:**
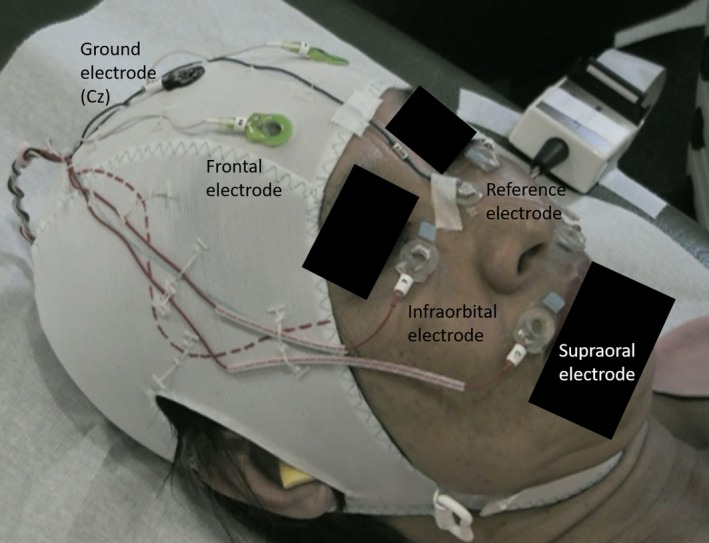
Custom‐made cap for superficial facial electromyography recording during rsfMRI scanning

To detect involuntary facial movements, which possibly confound the resting‐state signal, we recorded surface EMG measurements of the facial muscles simultaneously to the MRI scanning. We used a custom‐made, MRI‐compatible cap with six bilateral monopolar surface electrodes applied to the occipitofrontalis, orbicularis oculi, and orbicularis oris muscles with a reference electrode on the nose and a ground electrode at the vertex (Cz) (Easycap, Herrsching, Germany; Figure 1).

An MR‐compatible, optical cable transferred the EMG signal after amplification and A/D conversion (Brain Products GmbH, Gilching, Germany) from the scanner room to a PC, which recorded the EMG data using the Vision Recorder Software (Brain Products GmbH, Gilching, Germany). The online EMG measurement was synchronized to the rsfMRI scans by volume trigger signals of the MR scanner.

The subjects were asked to keep their eyes closed during MR data acquisition. For quality control, we performed an additional EMG measurement outside the scanner, while the subject's face was videotaped under different conditions: at rest with eyes closed (2 min, dimmed light) and open (1 min, dimmed light) as well as during intentional blinking, ocular movement, swallowing, smiling, nose wrinkling, and unilateral lifting of the corner of the mouth.

EMG data analysis was performed offline with the Brain Vision Analyzer Software 2.0.1 (Brain Products GmbH, Gilching, Germany). After correction for MR gradient and cardioballistic artifacts as implemented in the software, the data were analyzed with regard to the blinking frequency in a semiautomatic mode using independent component analysis (ICA). The EMG recording outside the scanner during rest was used for reference, as the comparison with the videotape made an attribution of certain signal changes to eyeblinks or ‐movements possible. This procedure was used to individualize the ICA parameters for each subject, which was used afterward for the analysis of the EMG data that had been recorded during the MRI scan. The results of the ICA were verified and if necessary corrected by visual inspection of the EMG signal during the MRI scanning (Table 1).

### 2.4 MRI data acquisition

MRI data were collected during rest with an 8‐channel‐head coil on a 3T‐Achieva MR scanner (Philips, the Netherlands) using a gradient T2*‐weighted echo‐planar image (EPI) sequence (TR/TE = 2,142/30 ms, voxel size 3 × 3 × 3 mm^3^, 42 slices, FoV 216 × 204 × 126 mm, scan time 11 min, 300 images per scan). A high‐resolution T1‐weighted structural image was acquired using a three‐dimensional gradient‐echo sequence for anatomical reference.

### 2.5 MRI data analysis

Imaging data were preprocessed using Statistical Parametric Mapping version 12.0 (SPM12, http://www.fil.ion.ucl.uk/spm) based on MATLAB (MathWorks, Natick, USA). After realignment of the functional images in order to correct for head motion, they were coregistered to the structural image and normalized to the Montreal Neurologic Institute (MNI) template using SPM8. Normalized images were smoothed with an isotropic Gaussian kernel of 8 mm FWHM. For quality control of head motion, a 1.5 mm absolute head translation was applied as threshold for exclusion of volumes (Table S1).

A region of interest (ROI)‐based analysis of resting‐state FC was conducted using the CONN toolbox (v15a) (Whitfield‐Gabrieli & Nieto‐Castanon, 2012), which analyzes the low‐frequency temporal correlations of blood oxygenation level‐dependent (BOLD) signals during rest between seed ROIs and all other voxels of the brain and generates ROI‐specific spatial FC maps. Considering previous imaging literature in dystonia, we selected the following bilateral seed ROIs from the AAL‐atlas of the WFU PickAtlas‐toolbox putatively involved in the pathophysiology of the disorder (http://www.nitrc.org/projects/wfu_pickatlas): caudate, anterior, middle and posterior cingulum, insula, putamen, pallidum, thalamus, pre‐ and postcentral gyrus, premotor cortex, supplementary motor area (SMA), and 11 cerebellar ROIs including dentate and (as unilateral ROI) vermis. A regression with several confounders was applied to the single‐subject data, namely the six realignment parameters computed from image preprocessing, the time series of averaged CSF signal and the averaged white matter signal. This multiple regression model reduces signal fluctuations unlikely to be related to FC. The averaged BOLD time series was obtained from each seed ROI after temporal band‐pass filtering (0.01 Hz < *f* < 0.1 Hz).

First‐level analysis was performed correlating the mean time course from the seed ROI to whole brain voxels creating connectivity maps for each seed region. These connectivity maps served to group‐level analyses comparing differences in connectivity among patients before BTX treatment (pre‐BTX) versus HCs and patients after BTX treatment (post‐BTX) versus HCs. Additionally, we performed a paired *t* test for the comparison between patients before and after treatment with BTX, as provided by the CONN toolbox. A cluster defining threshold of p < 0.001 uncorrected was applied to perform a family‐wise error (FWE) correction of p < 0.05 on the cluster level (one‐sided) in order to avoid false‐positive results (Eklund, Nichols, & Knutsson, 2016).

To test for correlations between the severity of dystonia and FC changes, we extracted average connectivity values for individual patients of each suprathreshold cluster resulting from the between‐group comparisons of ROI‐whole brain FC pre‐BTX versus HCs. These values were reentered as a second‐level covariate and correlated to the pre‐BTX‐JRS and pre‐BTX‐BDS scores using the CONN toolbox. The coefficient of determination (R^2^) was extracted for each correlation test.

## 3 RESULTS

### 3.1 Clinical data

There was no significant difference in age between patients and HCs. The JRS sum score decreased from pre‐BTX to post‐BTX, though without significant difference between both states (*p* = .41). Similarly, the patients’ rating using the BDS improved (not reaching significance) after treatment with BTX (Table 1).

### 3.2 EMG results

The analysis of the online recorded EMG signals with closed eyes revealed no significant difference regarding the eyelid muscle activity between patients and controls (*p* = .11) (Table 1).

### 3.3 Resting‐state fMRI

#### 3.3.1 FC comparison between patients’ pre‐BTX and HCs

**Figure 2:**
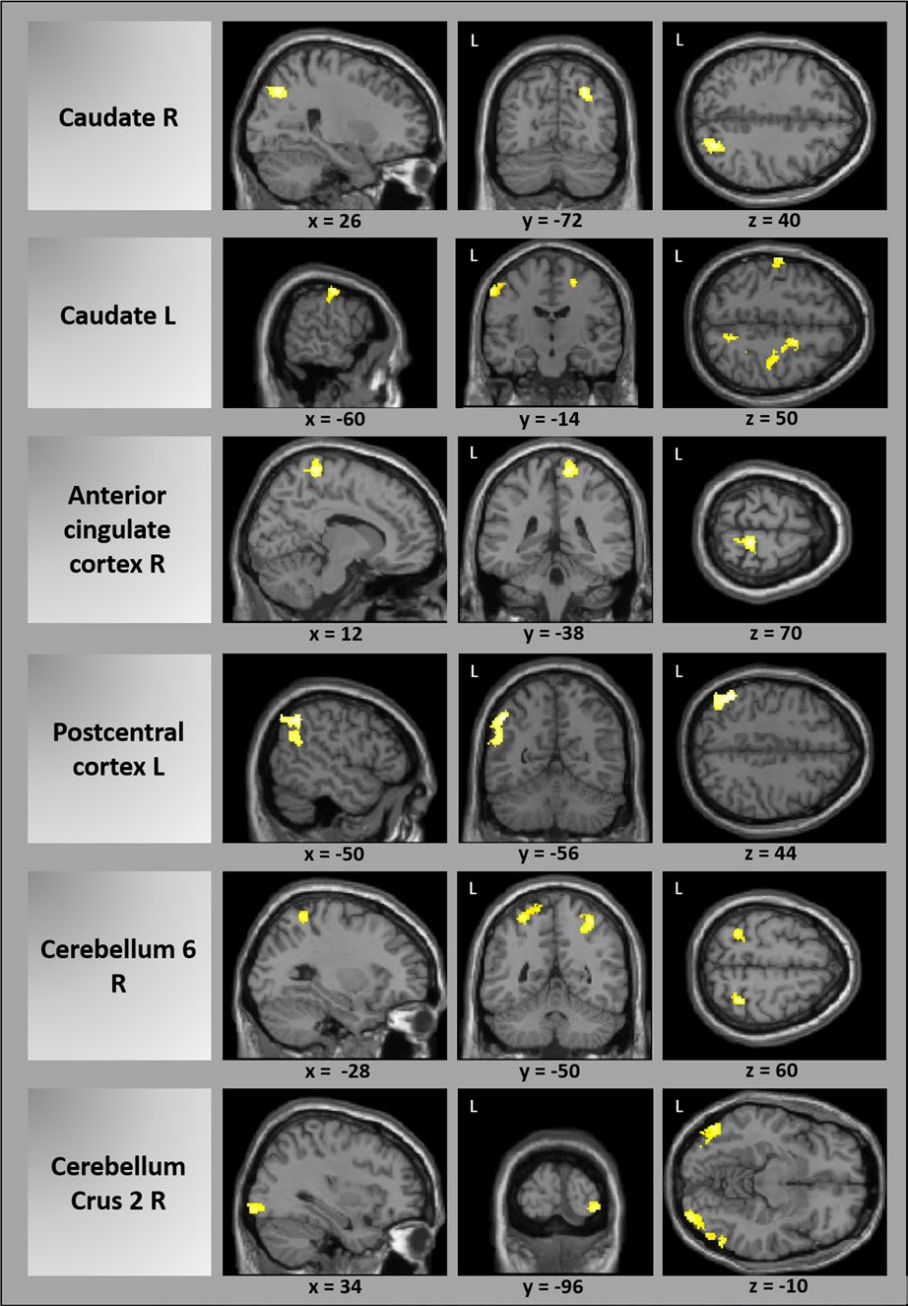
Reduced functional connectivity in patients before botulinum toxin treatment versus healthy controls exemplarily for several seed ROIs (cluster p‐FWE‐corrected). A complete list of all results can be found in Table 2. L, left; R, right

The patient group showed reduced connectivity in 16 of 45 regions that were used as seeds in the seed‐to‐voxel connectivity analysis in comparison with HCs (Table 2). FC was reduced between the left caudate seed and primary somatosensory cortex, primary motor, premotor, and supplementary motor cortex, and primary, secondary, and associative visual cortices. Furthermore, FC was decreased between both, the right and the left caudate with the right parietal association cortex and between the left putamen and the right supramarginal gyrus and insular cortex, superior/inferior temporal lobe, and middle and ventral anterior cingulate cortex. A FC reduction was also found between the right anterior cingulate cortex with bilateral primary somatosensory, right primary motor cortex, and the left supramarginal cortex; between the right middle cingulate cortex and the left insular and auditory cortex; between the left middle cingulum and left supramarginal gyrus; and between the left posterior cingulum seed and the right primary somatosensory and motor/premotor cortex. FC was reduced between the right and left insula. FC was also decreased between each postcentral cortex and the left supramarginal and angular gyrus, and of the right postcentral cortex to the right anterior cingulum, left parietal association cortex (BA 7), and right cerebellum. Bilateral premotor cortices showed reduced FC to the right premotor and primary somatosensory and motor cortex and to the left supramarginal gyrus. FC of the right cerebellum (area 6) to the bilateral parietal association cortex and the left somatosensory and visual association cortex was decreased as well as FC between the right cerebellar crus 2 seed and bilateral secondary and associative visual cortices and between left cerebellar crus and cerebellar area 8. A FC reduction was found between vermis and the left paracentral lobule. No FC increase in patients compared with controls was found for any of the seed ROIs (Table 2, Figure 2).

**Table 2:** Reduced functional connectivity in patients before BTX treatment versus healthy controls (Pre‐BTX vs. HCs)

Seed ROIs (AAL‐atlas)	Targets	Functional areas	Peak MNI coordinate (*x y z*)	Cluster size (voxel)	*p*
Caudate L	Postcentral, precentral, and superior frontal gyrus L	Primary somatosensory, premotor, and supplementary motor cortex (BA 3, 6)	−60 −14 +50	193	.0190
	Superior parietal lobule R	Parietal association cortex (BA 7)	+14 −62 +54	156	.0472
	Superior and middle occipital lobe L	Primary, secondary and associative visual cortex (BA 17, 18, 19)	−18 −76 +06	310	.0014
	Inferior occipital and temporal lobe, fusiform gyrus R	B 37	+36 −60 −04	195	.0181
	Superior and middle occipital and parietal lobe R	Parietal association cortex (BA 7)	+32 −68 +30	154	.0497
	Precentral, postcentral and superior frontal lobe R	Primary somatosensory, primary motor, premotor, and supplementary motor cortex (BA 3, 4, 6)	+32 −22 +54	445	.0001
Caudate R	Superior Occipital cortex and superior parietal lobule R	Parietal association cortex and associative visual cortex (BA 7, 19)	+26 −72 +40	306	.0017
Putamen L	Inferior temporal lobe, fusiform gyrus R	BA 37	+44 −58 −08	435	.0001
	Superior temporal lobe, insula and supramarginal gyrus R	Insula, auditory, and parietal association cortex (BA 13, 40–42)	+50 −34 +18	584	.0000
	Middle and ventral anterior cingulate cortex R	BA 24	+16 −12 +40	147	.0424
Cingulate gyrus, anterior part R	Postcentral and precentral gyrus R	Primary motor and somatosensory cortex (BA 3, 4)	+12 −38 +70	171	.0374
	Postcentral, supramarginal and superior temporal gyrus L	Primary somatosensory, auditory and insular cortex, and parietal association cortex (BA 2, 3, 13, 40–43)	−62 −20 +10	795	.0000
Cingulate gyrus, mid part L	Supramarginal gyrus L	Parietal association cortex (BA 40)	−54 −44 +28	357	.0012
Cingulate gyrus, mid part R	Insula, Heschl Gyrus, and Rolandic operculum L	Insular and auditory cortex (BA13, 41)	−40 −28 +10	239	.0092
Cingulate gyrus, posterior part L	Precentral and postcentral gyrus R	Primary somatosensory and motor, premotor and supplementary motor cortex (BA 3, 4, 6)	+34 −14 +66	665	.0000
Insula R	Planum temporale, Heschl gyrus, and insula	Auditory and insular cortex (BA 13, 41)	−44 −34 +12	180	.0385
Postcentral gyrus L	Inferior parietal lobule, supramarginal and angular gyrus L	Parietal association cortex (BA 39, 40, 7)	−50 −56 +44	505	.0001
Postcentral gyrus R	Cerebellum Crus 1 and 2, Cerebellum 6 R		+26 −62 −28	502	.0001
	Superior and inferior parietal lobule, supramarginal gyrus and angular gyrus L	Parietal association cortex (BA 7, 39, 40)	−44 −62 +44	684	.0000
	Dorsal anterior cingulate and dorsolateral prefrontal cortex R	BA 9, 32	+12 + 36 + 24	239	.0123
Premotor gyrus L	Angular and supramarginal gyrus L	Parietal association cortex (BA 39,40)	+36 −06 +64	505	.0059
	Precentral and postcentral gyrus R	Primary somatosensory and motor, premotor and supplementary cortex (BA 1–4, 6)	+64 –16 + 42	235	.0123
Premotor gyrus R	Postcentral and precentral gyrus R	Primary somatosensory and motor, premotor and supplementary cortex (BA 1–4, 6)	+64 −26 +44	325	.0016
	Postcentral and supramarginal gyrus L	Primary somatosensory and parietal association cortex (BA 2, 40)	−48 −28 +30	246	.0079
Cerebellum 6 R	Superior and inferior parietal lobule R	Parietal association cortex und parietal association cortex (BA 7, 40)	+34 −48 +58	307	.0021
	Superior parietal lobule L	Somatosensory and parietal association cortex (BA 5, 7)	−28 −50 +60	169	.0412
	Inferior and middle occipital lobule L	Associative visual cortex (BA 19)	−40 −72 −10	183	.0296
Cerebellum Crus 1 L	Cerebellum 8 L		−32 −42 −46	251	.0130
Cerebellum Crus 2 R	Inferior and middle occipital lobule L	Secondary and associative visual cortex (BA 18, 19)	−52 −76 −10	268	.0082
	Inferior and middle occipital lobule and inferior temporal, fusiform gyrus R	Secondary and associative visual cortex (BA 18, 19, 37)	+34 −96 −10	381	.0011
Vermis	Paracentral lobule L	Somatosensory association and primary motor cortex (BA 4, 5)	−16 −38 +56	208	.0154

Peak MNI coordinates of regions with reduced FC to a seed ROI (AAL‐atlas; *p* < .05, cluster p‐FWE‐corrected for multiple comparisons).

AAL, automated anatomic labeling; BA, Brodmann area; BTX, botulinum toxin; HC, healthy control; FWE, family‐wise error; L, left; MNI, Montreal Neurological Institute; pre‐BTX, before BTX treatment; R, right; ROI, region of interest; vs., versus.

There was a positive correlation between BDS and the FC reduction between the right cerebellum crus 2 and the right secondary and associative visual cortex (*r* = .65), which corresponds to a negative correlation of disease severity and FC reduction. Beyond that, there was no significant correlation between any of these FC results and the severity of the dystonic symptoms (JRS and BDS).

#### 3.3.2 FC comparison between patients’ post‐BTX and HCs

After treatment with BTX, FC was reduced in patients in comparison with HCs in six of the 45 regions which were used as seeds in the seed‐to‐voxel connectivity analysis (Table S2). It was decreased between the left caudate and the right dorsolateral prefrontal cortex as well as between the right pallidum and the right cerebellum, between the left middle cingulum and the left supramarginal gyrus, and between the right middle cingulum seed and the left superior temporal gyrus and premotor and supplementary motor cortex. Furthermore, FC was reduced between the left postcentral cortex and left supramarginal gyrus and auditory and insular cortex as well as between left‐sided cerebellar ROIs with the right supramarginal gyrus and auditory, insular and primary somatosensory cortex. FC was increased for two seeds, namely between the right thalamus and right orbitofrontal areas and between the right‐sided cerebellar area 4/5 and the right associative visual cortex (Table S2).

#### 3.3.3 Modulation of FC by BTX treatment

The comparison of the FC in patients before and after BTX treatment showed an increased FC for only one of the 45 seeds after treatment, specifically for the right cerebellar region 7b with the right associative visual cortex and fusiform gyrus (Table 3a). FC was reduced following BTX injection for six regions that were used as seeds of the seed‐to‐voxel‐connectivity analysis (Table 3b): Between the left pallidum and left cerebellum (area 4/5 and 6), between the right pallidum and bilateral caudate, left pallidum and left putamen, between the right thalamus and the right middle cingulum and supplemental motor areas, between the left posterior cingulum and left‐sided cerebellar regions as well as between left cerebellar regions and the bilateral frontal eye field, left parietal association, associative visual, dorsolateral prefrontal, premotor and supplementary motor cortex, and left middle frontal and temporal gyrus (Table 3).

**Table 3:** (a) Reduced functional connectivity in patients after versus before BTX treatment (Post‐BTX vs. Pre‐BTX). (b) Increased functional connectivity in patients after versus before BTX treatment (Post‐BTX vs. Pre‐BTX)

Seed ROIs (AAL‐atlas)	Targets	Functional areas	Peak MNI coordinate (*x y z*)	Cluster size (voxel)	*p*
(a)					
Pallidum L	Cerebellum 4 5, 6 L		−20 −56 −26	209	.0018
Pallidum R	Caudate L > R, Pallidum L and Putamen L		−02 +12 +02	233	.0004
Thalamus R	Cingulate gyrus (mid part) and supplemental motor area R	Ventral anterior and dorsal posterior cingulate cortex; premotor and supplementary motor cortex (BA 6, 24, 31)	+10 −10 +40	141	.0235
Cingulate gyrus, posterior part L	Cerebellum 4 5, 3 and brainstem L		−06 −34 −12	139	.0298
Cerebellum 3L	Superior and middle frontal gyrus R	Frontal eye field (BA 8)	+22 + 46 + 54	169	.0077
Cerebellum 10 L	Middle and superior frontal gyrus L	Frontal eye field, dorsolateral prefrontal cortex, premotor and supplementary motor cortex (BA 6, 8, 9)	−42 +14 +38	840	.0000
	Superior and inferior parietal lobule, angular and supramarginal gyrus and middle occipital cortex L	Parietal association cortex, associative visual cortex (BA 7, 19, 39, 40)	−38 −66 +48	574	.0000
	Middle and inferior frontal gyrus L	Orbitofrontal area (BA 11, 47)	−40 +40 −12	267	.0003
	Middle and inferior temporal gyrus L	Middle temporal gyrus (BA 21)	−64 −34 −14	137	.0187
(b)					
Cerebellum 7b R	Inferior temporal gyrus and middle occipital gyrus R, fusiform gyrus R	Associative visual cortex (BA 37)	+60 −68 −10	174	.0207

Peak MNI coordinates of regions with reduced FC to a seed ROI (AAL‐atlas; p < 0.05, cluster p‐FWE‐corrected for multiple comparisons).

AAL, automated anatomic labeling; BA, Brodmann area; BTX, botulinum toxin; HC, healthy control; FWE, family‐wise error; L, left; MNI, Montreal Neurological Institute; pre‐BTX, before BTX treatment; post‐BTX, after BTX treatment; R, right; ROI, region of interest; vs., versus.

## 4 DISCUSSION

Patients with blepharospasm or mild OFD showed a widespread pattern of altered FC in connections from and to basal ganglia, primary and secondary sensorimotor cortices, parietal association cortices, visual areas, and cerebellum. This changed after BTX treatment.

The basal ganglia are considered as the central network nodes in dystonia (Neychev et al., 2011; Niethammer, Carbon, Argyelan, & Eidelberg, 2011). In our study, the FC of the caudate nucleus seemed to be most affected in patients. FC was reduced between the caudate and the parietal association cortex, primary somatosensory cortex, and primary and secondary motor cortices as well as visual cortices in patients before BTX treatment. Furthermore, we found a reduced FC between putamen and the anterior cingulate cortex and parietal association cortex.

These results are congruent with the known affection of striato‐pallido‐thalamo‐cortical loops in other forms of dystonia. Two rsfMRI studies in blepharospasm found local changes of brain activity in putamen, pallidum, and thalamus (Yang et al., 2013; Zhou et al., 2013). ^18^F‐FDG‐PET‐studies demonstrated an altered metabolism in striatum and thalamus (Emoto et al., 2010; Esmaeli‐Gutstein, Nahmias, Thompson, Kazdan, & Harvey, 1999; Kerrison et al., 2003; Suzuki et al., 2007). Activation of the caudate was increased in patients with OFD during tactile stimulation in the face (Dresel et al., 2011). Other fMRI studies showed an increased activation of putamen and thalamus during blinking in blepharospasm (Baker et al., 2003; Schmidt et al., 2003). Voxel‐based morphometry (VBM) and diffusion tensor imaging (DTI) showed changes of gray or white matter in putamen, thalamus, and caudate (Black, Ongur, & Perlmutter, 1998; Etgen, Muhlau, Gaser, & Sander, 2006; Obermann et al., 2007; Yang et al., 2014).

The reduced FC between caudate and motor cortices might contribute to impaired motion control leading to the characteristic involuntary movements, as basal ganglia are important for the selection and inhibition of motor commands (Neychev et al., 2011). The same accounts for reduced FC of the putamen to the anterior cingulate cortex, a region which is relevant for motor planning and error correction (Arrighi et al., 2016). In this context, the anterior cingular cortex as seed ROI showed altered FC with the primary motor cortex and the posterior cingulate cortex with the premotor, primary, and supplementary motor cortex. Premotor cortex showed changed FC to the primary motor cortex. Previously, fMRI in OFD patients showed reduced activation of the premotor and primary motor cortex during a whistling task (Dresel et al., 2006), and activation in the rostral cingulate motor area was increased during voluntary eye spasms in blepharospasm patients (Baker et al., 2003). Glucose metabolism was described to be increased in posterior and anterior cingulate cortex (Kerrison et al., 2003). VBM showed alterations of gray matter volume or density in the primary sensorimotor cortex and (anterior) cingulum (Horovitz, Ford, Najee‐Ullah, Ostuni, & Hallett, 2012; Martino et al., 2011; Suzuki, Kiyosawa, Wakakura, Mochizuki, & Ishii, 2011).

Beside the motor system, FC of primary and secondary somatosensory cortices was also found to be changed. FC was reduced between the primary somatosensory cortex and anterior cingulate cortex, between parietal association cortex and cerebellum, and between premotor cortices and primary somatosensory cortex as well as parietal association cortex. FC between the caudate, primary somatosensory cortex, and cerebellar seed ROIs to the parietal association cortex as well as between cingular seeds and primary and associative somatosensory cortices was diminished.

Clinically, the somatosensory system is considerably affected in dystonia in general and especially in blepharospasm where sensory tricks like touching the eyelids or purpose‐built spectacles lead to an improvement. Many patients complain about sensory phenomena ‐like photophobia or a burning sensation (Hallett, Evinger, Jankovic, Stacy, & Workshop, 2008). Electrophysiologically, deficits in temporally discriminating sensory stimuli have been demonstrated in blepharospasm (Fiorio et al., 2008) and appear to be due to a loss of inhibition (Hallett et al., 2008). One of the aforementioned fMRI studies reported an overactivity of the postcentral gyrus in OFD patients during a whistling task (Dresel et al., 2006). During tactile stimulation in the face, the activation in primary and secondary cortex was deficient. Both findings could not be reversed by clinically effective BTX treatment pointing to a possible causative role of a dysfunction within the somatosensory system in OFD rather than being only a secondary epiphenomenon (Dresel et al., 2011). Based on these and other studies which demonstrated sensory deficits also in clinically unaffected body areas, dystonia has no longer been regarded as pure motor disorder, but as a sensory‐motor disorder (Avanzino, Tinazzi, Ionta, & Fiorio, 2015).

Previous studies examining changes of grey matter density or white matter architecture in patients with OFD reported changes in areas being close to our results of FC changes and therefore further support the notion of the relevant contribution of the somatosensory system to the pathophysiology of OFD: An increased bilateral gray matter density of the sensorimotor cortex and of the left cingulate gyrus was reported (Martino et al., 2011). Another VBM study showed an increased gray matter volume within the right somatosensory cortex (Horovitz et al., 2012). Furthermore, a decrease of gray matter in the left inferior parietal lobe in blepharospasm was found (Etgen et al., 2006). The diversity of these findings might be explained methodologically by differences in MRI sequences (Hallett et al., 2008). However, the location of cortical volume alterations seems to be more important and also more consistent than the direction of volume changes (Hallett et al., 2008). Besides, fractional anisotropy as measure of white matter integrity was decreased in the superior parietal lobe in a DTI study (Yang et al., 2014).

Functionally, FC alterations between premotor, cingulate, and postcentral cortices as well as between postcentral cortex and parietal association cortex and the change of striatal connectivity to primary and associative somatosensory cortices in our study fit with the concept of impaired sensorimotor integration in dystonia, that is, the interaction of the sensory system with the motor system (Quartarone & Hallett, 2013).

Reduced FC of different cerebellar ROIs with the secondary and associative visual cortex, somatosensory association cortex and parietal association cortex support the importance of the cerebellum in the pathophysiology of blepharospasm/OFD (Shakkottai et al., 2016).

Altered glucose metabolism was reported in the cerebellum at rest in blepharospasm (Hutchinson et al., 2000; Kerrison et al., 2003; Suzuki et al., 2007). An increase of functional activity during blinking was reported in the cerebellum along with the primary and associative visual cortices more extensively in blepharospasm patients than in HCs (Baker et al., 2003; Schmidt et al., 2003). Reductions of fractional anisotropy in the anterior cerebellar lobe were demonstrated in blepharospasm (Yang et al., 2014) similar to changes of cerebello‐thalamic tracts in genetic dystonia (Argyelan et al., 2009; Carbon, Kingsley, Tang, Bressman, & Eidelberg, 2008). The cerebellum is involved in the processing of proprioceptive inputs and temporal and spatial discrimination and influences somatosensory thresholds in the cortex (Lehericy, Tijssen, Vidailhet, Kaji, & Meunier, 2013). Again, the FC change between cerebellar and associative somatosensory regions might be relevant for impaired sensorimotor integration in dystonia.

FC alterations between cerebellum and visual cortices in our study might also be seen in the context of an altered modulation of sensoric (i.e., visual) information and visuomotor integration. Together with the reduced FC from the basal ganglia, this might point to a role of functional alterations in visual cortices in blepharospasm. This is plausible in the context of photophobia and the finding that sunlight exposure or eye diseases might be risk factors for the disease (Defazio et al., 2011; Molloy et al., 2015). Blepharospasm can markedly impair vision up to a functional blindness, which might result in altered feedback signals. The negative correlation of the FC reduction with the patients’ evaluation of disease severity might suggest that the FC change between cerebellum and visual cortex is a secondary phenomenon in the sense of a compensational mechanism. Increased negative FC of the cerebellum with the visual cortex has also been shown for patients with writer's cramp (Dresel et al., 2014). Recently, alterations in FC between the cerebellar and visual network were found in musicians with embouchure dystonia and have been interpreted as cerebellar deficit of visuomotor and visuotactile integration (Haslinger et al., 2016). Visual cortices had not been included in our primary set of seed ROIs, but our results comprise significant changes of FC to visual cortices as targets of several seed ROIs. This underlines their possible functional role (be it primary or secondary) in blepharospasm.

It is still unclear to which extent changes in brain function can be modulated by peripheral muscle denervation with BTX. Some electrophysiological results implicate a BTX‐induced cortical plasticity, which has been thought to result from changes in afferent input by denervation of intrafusal fibers (Byrnes et al., 1998; Ceballos‐Baumann, Sheean, Passingham, Marsden, & Brooks, 1997; Gilio et al., 2000) or from an altered proprioceptive feedback from paretic muscles (Gilio et al., 2000). Indication for such a central effect also came from the studies in OFD mentioned above, where BTX therapy modulated movement‐induced premotor activation (Dresel et al., 2006) as well as thalamic and putaminal activation during somatosensory stimulation (Dresel et al., 2011).

Our data point to a change of FC in patients following BTX treatment, which increased FC between cerebellum and associative visual cortex. FC was reduced by BTX between cingular and cerebellar ROIs, between thalamus and cingulum and supplemental motor areas and between cerebellum and frontal eye field, parietal association cortex, and temporal gyrus. As a limitation, the treatment with BTX had no significant effect on objective and subjective ratings of disease severity, which demonstrated only a trend for clinical improvement. This is congruent with the experience that the clinical effect in patients with long‐term BTX treatment often is less impressive than after the first treatment sessions as patients reach some kind of “equilibrium” with regular quarterly injections and do not wait with the next treatment until the BTX effect has noticeably waned.

We performed simultaneous electromyographic recording of facial movements, which showed no significant differences of eye blink frequency between patients and HCs. Thus, even though due to technical limits of online EMG recording, we cannot completely rule out subclinical muscle activity, and this makes it very unlikely that FC differences between patients and controls are due to differences in periocular muscle activity.

At the end, functional imaging does not allow to decide if the results reflect primary, secondary, or compensatory phenomena. The change of FC does not give information about the direction of information transfer, and it is not clear, how far task‐related imaging results that we discussed above can be transferred to the interpretation of time–course correlations as measured in rsfMRI. Resting‐state FC reflects functional communication between brain areas, even though an anatomical connection might not be known, for example, for contralateral supratentorial connections or ipsilateral infra‐ to supratentorial connections. The question why some connections show increased, others decreased FC must remain unanswered. Our results include many regions and connections, which might be attributed to the network character of dystonia. Our approach aimed at characterizing FC within this network by choosing a large set of seed ROIs without overly restricting our a priori hypotheses. On the other hand, in some cases, this makes a convincing interpretation for every single connection difficult.

The changes of functional connections in blepharospasm/OFD show considerable overlap with previous resting‐state imaging studies in other types of focal dystonia. More recently, rsfMRI in focal dystonias revealed FC changes in cerebello‐basal ganglia‐thalamo‐cortical loops in writer's cramp (Dresel et al., 2014) and cervical dystonia (Delnooz, Pasman, Beckmann, & van de Warrenburg, 2013, 2015). In embouchure dystonia, FC was changed for motor, auditory, cerebellar, and visual networks, in laryngeal dystonia in sensorimotor and frontoparietal networks (Battistella, Fuertinger, Fleysher, Ozelius, & Simonyan, 2016; Haslinger et al., 2016). Across different focal dystonias, abnormal network interactions were shown for basal ganglia, cerebellar, premotor, sensorimotor, and frontoparietal areas (Battistella, Termsarasab, Ramdhani, Fuertinger, & Simonyan, 2015). Our results suggest that this concept of dystonia as a network disorder might also be true for blepharospasm and OFD.

## CONFLICT OF INTERESTS

Related to this study: None. Other disclosures: A. Jochim has received travel grants from Ipsen Pharma GmbH, Merz Pharmaceuticals GmbH, Boston Scientific, and Julius‐Maximilians‐Universität Würzburg as well as speaker honoraria from Allergan. Y. Li has no conflict of interests. G. Gora‐Stahlberg has no conflict of interests. T. Mantel has received research support from the Kommission für Klinische Forschung (KKF, Klinikum rechts der Isar, Muenchen) and travel grants by Merz Pharmaceuticals GmbH and AbbVie Deutschland. M. Berndt has no conflict of interests. F. Castrop has no conflict of interests. C. Dresel has received research support from the Deutsche Forschungsgemeinschaft (DFG) and the Kommission für Klinische Forschung (KKF, Klinikum rechts der Isar, Muenchen). B. Haslinger has received research support from the Deutsche Forschungsgemeinschaft (DFG) and from Ipsen Pharma GmbH, has received speaker honoraria from Allergan and Bayer Health Care Pharmaceuticals, and has received travel grants from Ipsen Pharma GmbH.
