# Supplementary material for: Altered functional connectivity in blepharospasm/orofacial dystonia
Source: Brain Behav. 2017 Dec 18;8(1):e00894. doi: 10.1002/brb3.894 (PMC5853618; doi:10.1002/brb3.894)
Supplement: Supplementary file 1 [file BRB3-8-e00894-s001.docx]

**Supporting Information**

**Table S1:**

**Absolute head translation during scanning due to head movements**

|  | **x** | **y** | **z** |
| --- | --- | --- | --- |
| **mean** | 0.001 | 0.0065 | 0.0011 |
| **standard deviation** | 0.0017 | 0.0013 | 0.0012 |
| **maximum** | 0.0071 | 0.0097 | 0.0055 |
| **minimum** | 0 | 0.0035 | 0 |
| **median** | 0.0004 | 0.0061 | 0.0008 |

Head translation is shown for all subjects in mm.

**Table S2:**

**A: Reduced functional connectivity in patients after BTX treatment vs. healthy controls (PostBTX vs. HCs)**

| **Seed-ROIs**  (aal-Atlas) | **Targets** | **Functional areas** | **Peak MNI coordinate**  (x y z) | **Cluster size** (voxel) | **p** |
| --- | --- | --- | --- | --- | --- |
| **Caudate L** | Middle frontal gyrus R | dorsolateral prefrontal gyrus (BA 9, 46) | +54 +34 +30 | 162 | 0.0394 |
| **Pallidum R** | Cerebellum 7b, 8 R |  | +22 -74 -52 | 136 | 0.0432 |
| **Cingulate gyrus, mid part L** | Supramarginal gyrus L | parietal association cortex (BA 40) | -56 -44 +28 | 212 | 0.0174 |
| **Cingulate gyrus, mid part R** | Rolandic operculum, precentral and superior temporal gyrus | Premotor, supplementary motor and insular cortex L (BA 6, 13, 22) | -60 +08 +00 | 212 | 0.0171 |
| **Postcentral gyrus L** | Superior temporal lobe, insula and supramarginal gyrus R | parietal association, insular and auditory cortex (BA 13, 40, 41, 42) | +40 -20 +06 | 273 | 0.0060 |
|  | Superior temporal lobe and insula L | insular and auditory cortex (BA 13, 22, 41, 42) | -44 -36 +20 | 186 | 0.0359 |
| **Cerebellum 6 L** | Superior temporal lobe and supramarginal gyrus R | parietal association, auditory, insular and primary somatosensory cortex (BA 2, 13, 40-42) | +70 -20 +30 | 293 | 0.0001 |

**B: Increased functional connectivity in patients after BTX treatment vs. healthy controls (PostBTX vs. HCs)**

| **Seed-ROIs**  (aal-Atlas) | **Targets** | **Functional areas** | **Peak MNI coordinate**  (x y z) | **Cluster size** (voxel) | **p** |
| --- | --- | --- | --- | --- | --- |
| **Thalamus R** | Orbitofrontal and rectal gyrus R | orbitofrontal area (BA 11) | +10 +40 -26 | 286 | 0.0039 |
| **Cerebellum 4 5 R** | Cerebellum Crus 1 and 6, associative visual cortex and fusiform gyrus R | associative visual cortex (BA 19, 37) | +40 -66 -28 | 342 | 0.0007 |

Peak MNI coordinates of regions with reduced FC to a seed ROI (Aal-atlas; p < 0.05, cluster p-FWE-corrected for multiple comparisons).

AAL: automated anatomic labeling, BA: Brodmann area, BTX: botulinum toxin, HC: healthy control, FWE: family-wise error, L: left, MNI: Montreal Neurological Institute, postBTX: after BTX treatment, R: right, ROI: region of interest, vs.: versus
